# Evaluating *in vivo* data for drug metabolism and transport: lessons from Kirchhoff’s Laws

**DOI:** 10.3389/fphar.2024.1456677

**Published:** 2024-11-05

**Authors:** Leslie Z. Benet, Jasleen K. Sodhi

**Affiliations:** ^1^ Department of Bioengineering and Therapeutic Sciences, Schools of Pharmacy and Medicine, University of California San Francisco, San Francisco, CA, United States; ^2^ Department of Drug Metabolism and Pharmacokinetics, Septerna, South San Francisco, CA, United States

**Keywords:** metabolism, transport, pharmacokinetics, clearance, Kirchhoff’s laws

## Abstract

Mechanistic models of hepatic clearance have been evaluated for more than 50 years, with the first author of this mini-review serving as a co-author of the first paper proposing such a model. However, published quality experimental data are only consistent with the first of these models, designated as the well-stirred model, despite the universal recognition that this model is physiologically unrepresentative of what occurs with respect to liver metabolism and transport. Within the last 3 years, our laboratory has recognized that it is possible to derive clearance equations employing the concepts of Kirchhoff’s Laws from physics, independent of the differential equation approach that has been utilized to derive reaction rates in chemistry. Here we review our published studies showing that the equation previously believed to be the well-stirred model, when hepatic basolateral transporters are not clinically relevant, is in fact the general equation for hepatic clearance when only systemic drug concentrations are measured, explaining why all experimental data fit this equation. To demonstrate that the equations deriving the mechanistic models of hepatic elimination for the past 50 years are not valid, we show that when calculating Kp_uu_, the ratio of unbound drug concentration in the liver to the unbound concentration of drug in the systemic circulation, for the well-stirred, parallel tube and dispersion models, Kp_uu_ surprisingly can never exceed 1 and is a function of F_H_, the hepatic bioavailability following oral dosing. We believe that knowledgeable drug metabolism scientist and clinical pharmacologist will agree that this outcome is nonsensical.

## 1 Introduction

It is important to recognize that the limitations imposed by *in vivo* experimental and clinical studies must be considered when characterizing drug metabolism and transport data and their implications in drug dosing decisions. As we have recently summarized (Benet and Sodhi, 2024b), much of the pharmacokinetic analyses universally accepted by the field with respect to hepatic metabolism and transport are not consistent with the limitations of *in vivo* studies, where the only experimental measurements available are systemic drug concentrations and urinary excretion amounts as a function of time. These limitations apply to simple analyses of *in vivo* data using noncompartmental or compartmental pharmacokinetic models as well as physiologic based pharmacokinetic (PBPK) models. Decisions concerning drug dosing in a patient or changes in drug dosing due to disease states, pharmacogenomic and physiologic differences, and drug-drug interactions are all based on drug exposure, that is, area under the concentration-time curve (AUC). Thus, the relevance of the pharmacokinetic model is based on the ability to measure and/or predict exposure and changes in exposure. In this mini-review we limit our discussion to linear pharmacokinetic systems, but will address saturable processes in future publications, which are yet unpublished, although an abstract and poster describing this approach was presented at the 2024 International Society for the Study of Xenobiotics and Japanese Society for the Study of Xenobiotics (ISSX/JSSX) meeting, in Honolulu, HI, United States on September 16–18 (Benet and Sodhi, 2024c).

Systemic exposure and changes in systemic exposure due to metabolism and transport for linear systems are a result of dose, bioavailability (F) and clearance (CL), and changes in these three parameters as given in Equation 1 for a single dose over all time or for multiple doses at steady-state (ss) during a dosing interval.

$${AUC}_{0\to \infty or\;dosing\;interval\;at\;ss}=\frac{F\cdot Dose}{CL}$$ (1)

Changes in volume of distribution or rate constants do not change exposure unless they also result in clearance changes.

In the last 2 years we recognized that the derivation of clearance for *in vivo* drug metabolism and transport processes in series and in parallel could be simply accomplished independent of differential equations utilizing Kirchhoff’s Laws from physics (Patcher et al., 2022; Benet and Sodhi, 2023; Benet and Sodhi, 2024a; Benet and Sodhi, 2024b; Wakuda et al., 2024). In fact, we demonstrated that traditional pharmacokinetic equations based on differential equations for drug absorption and elimination utilized and taught for the past century cannot accurately define the measured AUC when clearance of drug from the administration site is comparable to or less than clearance of an iv bolus dose. Thus, using Kirchhoff’s Laws we can explain why it is possible to obtain bioavailability from systemic concentration measurements that exceed unity, why renal clearance can be a function of drug input processes, and why statistically different bioavailability measures may be found for urinary excretion versus systemic concentration measurements in the same study.

## 2 Kirchhoff’s laws applied to the pharmacokinetics of in series processes

We discovered, in 2022, that Kirchhoff’s Laws from physics would provide a pathway to derive clearance and overall rate constants for in series processes, independent of differential equation derivations (Patcher et al., 2022). We showed that consistent with Kirchhoff’s Laws for processes in series, the inverse of the overall clearance would equal the sum of the inverse of the individual rate-defining processes entering and the inverse of the individual rate-defining process leaving. Here for clearance as given in Equation 2

$$\frac{1}{{CL}_{overall}}=\frac{1}{{CL}_{entering\;rate-defining\;process}}+\frac{1}{{CL}_{leaving\;rate-defing\;process}}$$ (2)

A rate-defining process is one that, on its own, could potentially define a total clearance, and one that is possible to measure experimentally when it solely determines the clearance (e.g., liver blood flow). When the hepatic metabolic clearance (CL_H_) is derived and hepatic basolateral transporters are not clinically relevant, the entering rate-defining process is hepatic blood flow (Q_H_) and the leaving rate-defining process is the fraction unbound in blood (f_u,B_) multiplied by the hepatic intrinsic clearance (CL_int_) as given in Equation 3.

$$\frac{1}{{CL}_{H}}=\frac{1}{Q_{H}}+\frac{1}{f_{u,B}\cdot {CL}_{\mathrm{int}}}$$ (3)

When hepatic basolateral transporters are relevant to hepatic clearance, a third rate-defining process is included (Patcher et al., 2022), the difference between the intrinsic hepatic uptake clearance (CL_influx_) minus the intrinsic hepatic efflux clearance (CL_efflux_).

$$\frac{1}{{CL}_{H}}=\frac{1}{Q_{H}}+\frac{1}{f_{u,B}\cdot \left({CL}_{influx}-{CL}_{efflux}\right)}+\frac{1}{f_{u,B}\cdot {CL}_{\mathrm{int}}}$$ (4)

As we recently detailed (Benet and Sodhi, 2024b), Kirchhoff’s Laws are only applicable for rate-defining processes. That is a parameter that can by itself be a measurable result of *in vivo* experimental studies. Thus, when (CL_influx_ – CL_efflux_) is a positive value, it is possible that hepatic basolateral transport could be rate limited by this value. However, if (CL_influx_ – CL_efflux_) is a negative value, hepatic clearance could never equal this value, and the term should not be included in the CL_H_ equation (Equation 4), just as passive diffusion processes are not included in the CL_H_ equation. Hepatic clearance can never be defined by passive diffusion.

When a drug is dosed extravascularly (e.g., oral, subcutaneous, intramuscular, lymphatic, transdermal, inhalation), the entering rate-defining process is the clearance from the site of administration, a concept not previously considered in pharmacokinetics prior to our publications (Wakuda et al., 2024; Benet and Sodhi, 2024b), while the leaving rate-defining process is the clearance following an iv bolus dose as given in Equation 5.

$$\frac{1}{{CL}_{after\;extravascular\;dosing}}=\frac{1}{{CL}_{extravascular\;site}}+\frac{1}{{CL}_{iv\;bolus}}$$ (5)

## 3 Evaluating drug metabolism *in vivo* data

A very familiar equation results from rearrangement of Equation 3, which yields:

$${CL}_{H}=Q_{H}\cdot \frac{f_{u,B}\cdot {CL}_{\mathrm{int}}}{Q_{H}+f_{u,B}\cdot {CL}_{\mathrm{int}}}$$ (6)

**FIGURE 1:**
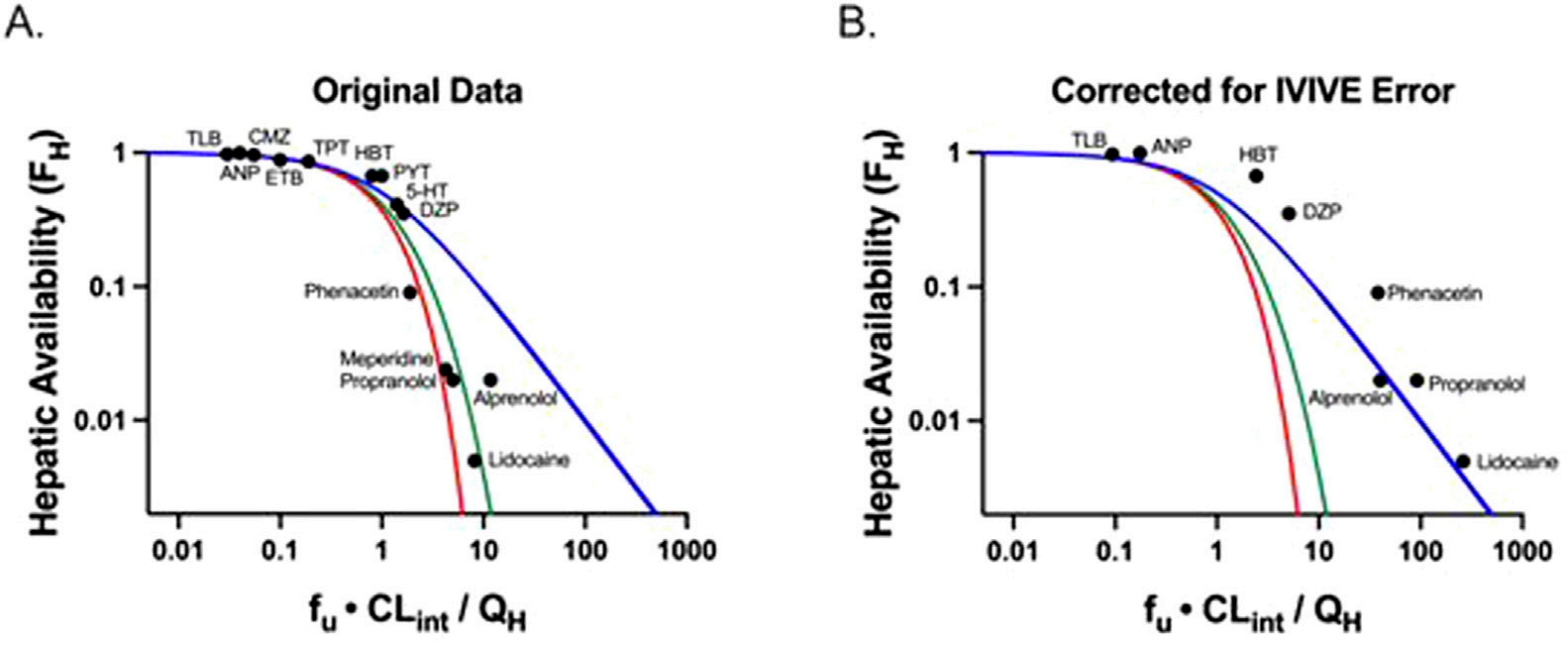
Adapted from Benet and Sodhi (2024b): Plots of hepatic availability (*F*_*H*_) vs. efficiency number (*f*_*u*_ *·CL*_*int*_ */Q*_*H*_) based on **(A)** originally published analysis, and **(B)** further corrected for in vitro-in vivo underprediction error. The theoretical clearance relationships are represented with lines in blue (the Equation 6 relationship; previously regarded as the well-stirred model), red (parallel tube model), and green (dispersion model). **(A)** Data points assuming no error in IVIVE prediction are depicted, based on original analysis from Roberts and Rowland (1986) and Iwatsubo et al. (1996). **(B)** Original data are corrected for degree of observed *in vitro* to *in vivo* (IVIVE) underprediction error, based on human liver microsomal IVIVE data reported by Wood et al. (2017). The five high extraction ratio compounds included in this analysis (alprenolol, lidocaine, meperidine, phenacetin and propranolol) are labeled. Additional compounds (low and moderate extraction ratio) are labeled with the following abbreviations: 5-HT, 5-hydroxytryptamine; ANP, antipyrine; CMZ, carbamazepine; DZP, diazepam; ETB, ethoxybenzamide; HBT, hexobarbitone; PYT, phenytoin; TLB, tolbutamide; TPT, thiopental. (Published with permission from Sodhi, J.S. *Identifying Xenobiotic Transporter Involvement in Complex Drug-Drug Interactions*, Doctoral Thesis, University of California San Francisco, 2020).

Equation 6 for the past half-century has been believed to be the well-stirred model (WSM). However, Equation 6 was derived making no assumptions concerning the mechanistic characteristics of hepatic elimination; it is simply the result of considering two in series steps in hepatic elimination utilizing Kirchhoff’s Laws. The universality of Equation 6 in defining hepatic clearance when only extrahepatic concentrations are measured is notably supported by the fact that all valid isolated perfused rat liver (IPRL) experimental studies, where only systemic concentrations are measured, are consistent with Equation 6 (Sodhi et al., 2020). There are no valid experimental studies that unambiguously demonstrate that IPRL data are better fit by the parallel tube model (PTM) or dispersion models (DMs) as compared to Equation 6. Of particular relevance to this previous statement is Figure 1A, which was presented by both Professors Rowland and Sugiyama as supporting the PTM and DM mechanisms of hepatic elimination at the September 2023 International Society for the Study of Xenobiotics (ISSX) symposium, “50 Years of Clearance Prediction.” Both speakers cited studies from their laboratories in presenting versions of Figure 1A (Roberts and Rowland, 1986; Iwatsubo et al., 1996), where the *y*-axis values are published hepatic availability *F*_*H*_ measures, experimentally determined from *ex-vivo* IPRL studies. Whereas the *x*-axis values are calculated efficiency numbers (*f*_*u*_ ⋅ *CL*_*int*_/*Q*_*H*_), which were determined by combining the experimentally utilized *Q*_*H*_ and *f*_*u*_ values from the IPRL study with a predicted *in vivo CL*_*int*_ that is based on in vitro-in vivo extrapolation (IVIVE) of *in vitro CL*_*int*_ measures from a different study. As we recently noted (Benet and Sodhi, 2024b) “Notably, the calculated *in vivo CL*_*int*_ values assume that IVIVE has no error, and that the *in vitro CL*_*int*_ value may accurately predict the *in vivo CL*_*int*_. In the last century, it may have been believed that IVIVE would give quantitatively accurate values, but we know today from multiple studies that this is not true and that throughout the field, as presently employed, IVIVE consistently underpredicts the *in vivo* measured experimental clearance values (Sodhi and Benet, 2021). At the time that Figure 1A was originally presented the authors understandably may not have appreciated this difference. But subsequently, both speakers have published with their colleagues that they recognize that the previous assumption of the accuracy of IVIVE is incorrect (Chiba et al., 2009; Rowland and Pang, 2018).

In Figure 1B, reproduced from Benet and Sodhi (2024b), we replotted the x-values for all of the data points where IVIVE error data were available using the degree of IVIVE underprediction for human microsome experiments, as reported by Wood et al. (2017). It is instructive that when the IVIVE underprediction is accounted for, all of the data appear to be best described by Equation 6 (blue line), previously regarded as the WSM. Thus, we maintain that although the field believes that the WSM is unphysiologic and that the PTM and DMs are more representative of liver elimination for high clearance drugs, there are no quality experimental studies available demonstrating that data are best described by the PTM and DMs when only systemic concentrations are measured. Equation 6 continues to best describe all valid experimental data available in the published literature.

## 4 Evaluating mechanistic models of hepatic elimination for *in vivo* data

As indicated above, using Kirchhoff’s Laws we showed that the equation previously believed to be the well-stirred model (Equation 6) could be derived independent of any mechanistic model. We maintain that when only systemic concentrations are measured, the correct relationship between hepatic clearance, hepatic blood flow and intrinsic hepatic clearance (when hepatic basolateral transporters are not clinically relevant) is Equation 6 and that Equation 6 is model-independent (i.e., it is not the well-stirred model). Sodhi et al. (2020) reanalyzed the published experimental IPRL data, the only data that can unambiguously differentiate the different mechanistic models of hepatic elimination and found that none of the studies support the parallel tube and dispersion models versus the fit to Equation 6.

However, if one chooses to ignore all experimental *in vivo* data, one can argue that our hypothesis is just a theoretical alternate proposal to the well-stirred model derivation (Rowland et al., 1973; Wilkinson and Shand, 1975). Yet recently we have demonstrated (Benet and Sodhi, 2024a) that the previously proposed mechanistic models of hepatic elimination, whether including or not including hepatic basolateral transport, lead to completely unrealistic relationships between Kp_uu_, the steady-state partition between unbound drug concentration in the liver and the unbound systemic blood concentration, and F_H_, the fraction of bioavailability due to hepatic clearance following oral dosing. Furthermore, we show that the proposal of Li and Jusko (2022) that when the unbound partition is measured between the drug concentration in the liver and the drug concentration in the blood exiting the liver leads to an even more preposterous outcome for Kp_uu_, with the result that Kp_uu_ equals 1.0 for all drugs when clearance is described by Equation 6. Thus, we assert that these additional confounding theoretical outcomes added to the finding that no experimental IPRL data support the parallel tube and dispersion models should convince our field that the models of hepatic elimination have no relevance when only systemic concentrations are measured.

As we point out (Benet and Sodhi, 2024a; Benet and Sodhi, 2024b), the error that has been made for the past 50 years is equating the steady-state (ss) rate of loss in the systemic circulation with the unbound concentration in the liver (C_Liver,u,ss_) multiplied by the intrinsic liver clearance (CL_Liver,int_) for each of the mechanistic models of hepatic elimination. Instead, the correct approach should be to multiply the concentration of total drug in the liver (C_Liver,ss_) by the liver clearance (CL_Liver_) as represented in Equation 7.

$${Rate\;of\;loss}_{ss}=C_{Blood,ss}\cdot {CL}_{Blood}=C_{Liver,ss}\cdot {CL}_{Liver}\neq C_{Liver,u,ss}\cdot {CL}_{Liver,\mathrm{int}}$$ (7)

We assert that if the drug clearance in the blood is rate limited by hepatic blood flow, should not the drug clearance in the liver also be rate limited by hepatic blood flow? Thus, we argue that there should be no basis in utilizing the final term of Equation 7 to further derive mechanistic models of hepatic elimination, and this error explains why experimental data also do not support any such derived hepatic disposition equations.

## 5 Evaluating *in vivo* drug transport data

### 5.1 The extended clearance model (ECM)

Up to the present, the incorporation of hepatic basolateral transport into hepatic clearance equations has been treated as an extension of the WSM, assuming that the inequality in Equation 7 is a valid equality, which has been presented as the extended clearance model (ECM) in a number of papers as we reviewed (Benet et al., 2018), giving Equation 8

$${CL}_{Blood,ECM}=\frac{Q_{H}\cdot {CL}_{influx}\cdot f_{u,B}\cdot {CL}_{\mathrm{int}}}{Q_{H}\cdot \left({CL}_{\mathrm{int}}+{CL}_{efflux}\right)+{CL}_{influx}\cdot f_{u,B}\cdot {CL}_{\mathrm{int}}}$$ (8)

Assuming that $\frac{{CL}_{influx}\cdot f_{u,B}\cdot {CL}_{\mathrm{int}}}{Q_{H}}\ll \left({CL}_{\mathrm{int}}+{CL}_{efflux}\right)$ , the familiar form of the ECM is given as Equation 9

$${CL}_{Blood,ECM}=\frac{{CL}_{influx}\cdot f_{u,B}\cdot {CL}_{\mathrm{int}}}{\left({CL}_{\mathrm{int}}+{CL}_{efflux}\right)}$$ (9)

Then the explanation for hepatic uptake to be the rate limiting step for statins and other acids with molecular weights greater than 400 is to assume that CL_efflux_ is negligible, so that the CL_int_ terms in the numerator and denominator may cancel, as presented by many colleagues, for example, by Sirianni and Pang (1997), Webborn et al. (2007), Kusuhara and Sugiyama (2009), Caminesch and Umehara (2012), Barton et al. (2013), and Varma et al. (2015).

There are four outcomes that demonstrate the deficiencies of the ECM (Equation 9). First, when clearance is assumed to be given by Equation 9, the value for Kp_uu_ determined at steady-state must always be less than F_H_, a completely unrealistic relationship (Benet and Sodhi, 2024a). Second, why should it be necessary for CL_efflux_ to be negligible for hepatic basolateral transport to be rate limiting? Couldn’t hepatic basolateral uptake be rate limiting as long as CL_influx_ was greater than CL_efflux_? Third, if hepatic basolateral uptake is the rate limiting process for hepatic clearance, it is not possible for CL_int_ to have any effect on hepatic clearance. And fourth, clearance incorporating hepatic basolateral transport (Equation 9) has only been derived for the WSM, not for the PTM and DMs.

### 5.2 Incorporating basolateral hepatic transport using Kirchhoff’s Laws

As first presented by Patcher et al. (2022), including hepatic basolateral transport in the general hepatic clearance equation (Equation 6) is given by:

$$\frac{1}{{CL}_{Blood}}=\frac{1}{Q_{H}}+\frac{1}{f_{u,B}\cdot {CL}_{\mathrm{int}}}+\frac{1}{f_{u,B}\cdot \left({CL}_{influx}-{CL}_{efflux}\right)}$$ (10)

Note that our approach to the relevance of influx and efflux at the hepatic basolateral border follows the same approach as universally used for secretion and reabsorption in the kidney, where it is the difference between the two processes that drives clearance, rather than separating out the two processes as is done in the ECM. Further, these parameters capture both the active plus the passive membrane passage clearances.

Then if $Q_{H}\gg f_{u,B}\cdot {CL}_{\mathrm{int}}\text{ and }f_{u,B}\cdot \left({CL}_{influx}-{CL}_{efflux}\right)$ solution of Equation 10 gives

$${CL}_{H}=\frac{f_{u,B}\cdot {CL}_{\mathrm{int}}\cdot \left({CL}_{influx}-{CL}_{efflux}\right)}{{CL}_{\mathrm{int}}+\left({CL}_{influx}-{CL}_{efflux}\right)}=\frac{f_{u,B}\cdot {CL}_{\mathrm{int}}}{1+\frac{{CL}_{\mathrm{int}}}{\left({CL}_{influx}-{CL}_{efflux}\right)}}=\frac{f_{u,B}\cdot \left({CL}_{influx}-{CL}_{efflux}\right)}{1+\frac{\left({CL}_{influx}-{CL}_{efflux}\right)}{{CL}_{\mathrm{int}}}}$$ (11)

From the Equation 11 relationship, when CL_int_ is much greater than $\left({CL}_{influx}-{CL}_{efflux}\right),$ hepatic basolateral transport is the rate limiting step; when $\left({CL}_{influx}-{CL}_{efflux}\right)$ is much greater that CL_int_, hepatic elimination is the rate limiting step. And Equation 11 defines the relationship at intermediate positions when both hepatic basolateral transport and hepatic elimination are relevant. All of the clinical clearance relationships for statins and other acids with molecular weights greater than 400 (i.e., Extended Clearance Classification System (ECCS) classes 1B and 3B drugs as defined by Varma et al., 2015) can be described by Equation 11.

## 6 Summary

When evaluating and predicting systemic concentrations for drugs undergoing hepatic metabolism and transport, we have proposed that a number of the clearance equations universally accepted for the past 50-years are not valid. This obviously is a very controversial proposal; however, we believe that experimental data strongly support our position. Here we review our published studies showing that what was previously believed to be the well-stirred model is in fact the general model for hepatic elimination when only systemic concentrations are measured, consistent with the finding that all quality experimental data only fit this relationship. We detail four reasons that the equation including basolateral transporter effects into the hepatic clearance equation, the Extended Clearance Model, is not valid. The correct equation derived from Kirchhoff’s Laws is consistent with all experimental data. Hepatic clearance equations when hepatic basolateral transporters are clinically relevant and when they are not relevant are simply derived based on Kirchhoff’s Laws independent of differential equation derivations.

## References

[B1] Barton H. A. Lai Y. Goosen T. C. Jones H. M. El-Kattan A. F. Gossed J. R. (2013). Model-based approaches to predict drug-drug interactions associated with hepatic uptake transporters: preclinical, clinical and beyond. Expert Opin. Drug Metab. Toxicol. 9, 459–472. 10.1517/17425255.2013.759210 23331046

[B2] Benet L. Z. Sodhi J. K. (2023). The uses and advantages of Kirchhoff’s Laws vs. differential equations in pharmacology, pharmacokinetics, and (even) chemistry. AAPS J. 25, 38. 10.1208/s12248-023-00801-w 37038013 PMC10832327

[B3] Benet L. Z. Sodhi J. K. (2024a). Are all measures of liver Kp_uu_ a function of F_H_, as determined following oral dosing, or have we made a critical error in defining hepatic drug clearance? Eur. J. Pharm. Sci. 196, 106753. 10.1016/j.ejps.2024.106753 38522769 PMC11813737

[B4] Benet L. Z. Sodhi J. K. (2024b). Commentary: pharmacokinetic theory must consider published experimental data. Drug Metab. Dispos. 52, 932–938. In press. Accepted for publication June 17, 2024. 10.1124/dmd.124.001735 38942444 PMC11331591

[B5] Benet L. Z. Sodhi J. K. (2024c). The application of Kirchhoff’s Laws to saturable, Michaelis-Menten, pharmacokinetics. Honolulu, HI: ISSX/JSSX Meeting. September, 2024, ISSX Online Abstracts, Suppl. 18 (1), Abstract P174.

[B23] Benet L. Z. Bowman C. M. Liu S. Sodhi J. K. (2018). The extended clearance concept following oral and intravenous dosing: theory and critical analyses. Pharm. Res. 35, 242. 30349948 10.1007/s11095-018-2524-0 PMC6364828

[B6] Caminesch G. Umehara K. (2012). Predicting human hepatic clearance from *in vitro* drug metabolism and transport data: a scientific and pharmaceutical perspective for assessing drug-drug interactions. Biopharm. Drug Dispos. 33, 179–194. 10.1002/bdd.1784 22407504

[B7] Chiba M. Ishii Y. Sugiyama Y. (2009). Prediction of hepatic clearance in human from *in vitro* data for successful drug development. AAPS J. 11, 262–276. 10.1208/s12248-009-9103-6 19408130 PMC2691463

[B8] Iwatsubo T. Hirota N. Ooie T. Suzuki H. Shimada N. Chiba K. (1996). Prediction of *in vivo* drug disposition from *in vitro* data based on physiological pharmacokinetics. Biopharm. Drug Dispos. 17, 273–310. 10.1002/(SICI)1099-081X(199605)17:4<273::AID-BDD961>3.0.CO;2-R 8845471

[B9] Kusuhara H. Sugiyama Y. (2009). *In vitro*-*in vivo* extrapolation of transporter-mediated clearance in the liver and kidney. Drug Metab. Pharmacokinet. 24, 37–52. 10.2133/dmpk.24.37 19252335

[B10] LiX.JuskoW. J. (2022). Assessing liver-to-plasma partition coefficients and *in silico* calculation methods: when does the hepatic model matter in PBPK? Drug Metab. Dispos. 50, 1501–1512. 10.1124/dmd.122.000994 36195337

[B11] Patcher J. A. Dill K. A. Sodhi J. K. Benet L. Z. (2022). Review of the application of Kirchhoff's Laws of series and parallel flows to pharmacology: defining organ clearance. Pharmacol. Ther. 239, 108278. 10.1016/j.pharmthera.2022.108278 36075300 PMC10832328

[B12] Roberts M. S. Rowland M. (1986). Correlation between *in-vitro* microsomal enzyme activity and whole organ hepatic elimination kinetics: analysis with a dispersion model. J. Pharmacol. 48, 177–181. 10.1111/j.2042-7158.1986.tb04540.x 2871151

[B13] Rowland M. Benet L. Z. Graham G. G. (1973). Clearance concepts in pharmacokinetics. J. Pharmacokinet. Biopharm. 1, 123–136. 10.1007/BF01059626 4764426

[B14] Rowland M. Pang K. S. (2018). Commentary on “The universally unrecognized assumption in predicting drug clearance and organ extraction ratio”. Clin. Pharmacol. Ther. 103, 386–388. 10.1002/cpt.921 29134634

[B15] Sirianni G. L. Pang K. S. (1997). Organ clearance concepts: new perspectives on old principles. J. Pharmacokinet. Biopharm. 25, 449–470. 10.1023/a:1025792925854 9561488

[B16] Sodhi J. K. Benet L. Z. (2021). Successful and unsuccessful prediction of human hepatic clearance for lead optimization. J. Med. Chem. 64, 3546–3559. 10.1021/acs.jmedchem.0c01930 33765384 PMC8504179

[B17] Sodhi J. K. Wang H.-J. Benet L. Z. (2020). Are there any experimental perfusion data that preferentially support the dispersion and parallel tube models over the well-stirred model of organ elimination? Drug Metab. Dispos. 48, 537–543. 10.1124/dmd.120.090530 32305951 PMC7289046

[B18] Varma M. V. Steyn S. J. Allerton C. El-Kattan A. F. (2015). Predicting clearance mechanism in drug discovery: extended clearance classification system (ECCS). Pharm. Res. 32, 3785–3802. 10.1007/s11095-015-1749-4 26155985

[B19] Wakuda H. Xiang Y. Sodhi J. K. Uemura N. Benet L. Z. (2024). An explanation of why dose-corrected area under the curve for alternate administration routes can be greater than for intravenous dosing. AAPS J. 26, 22. 10.1208/s12248-024-00887-w 38291293 PMC13115556

[B20] Webborn P. J. H. Parker A. J. Denton R. L. Riley R. J. (2007). *In vitro*-*in vivo* extrapolation of hepatic clearance involving active uptake: theoretical and experimental aspects. Xenobiotica 37, 1090–1109. 10.3109/00498250701557266 17968738

[B21] Wilkinson G. R. Shand D. G. (1975). Commentary: a physiological approach to hepatic drug clearance. Clin. Pharmacol. Ther. 18, 377–390. 10.1002/cpt1975184377 1164821

[B22] Wood F. L. Houston J. B. Hallifax D. (2017). Clearance prediction methodology needs fundamental improvement: trends common to rat and human hepatocytes/microsomes and implications for experimental methodology. Drug Metab. Dispos. 45, 1178–1188. 10.1124/dmd.117.077040 28887366

